# Statins enhances antitumor effect of oxaliplatin in KRAS-mutated colorectal cancer cells and inhibits oxaliplatin-induced neuropathy

**DOI:** 10.1186/s12935-023-02884-z

**Published:** 2023-04-17

**Authors:** Masanobu Tsubaki, Tomoya Takeda, Takuya Matsuda, Kana Kishimoto, Honoka Takefuji, Yuzuki Taniwaki, Misa Ueda, Tadafumi Hoshida, Kazufumi Tanabe, Shozo Nishida

**Affiliations:** 1 Division of Pharmacotherapy, Kindai University Faculty of Pharmacy, Kowakae, Higashi-Osaka, 577-8502 Japan; 2 Department of Pharmacy, Japanese Red Cross Society Wakayama Medical Center, Wakayama, Japan

**Keywords:** Colorectal cancer, Statin, Oxaliplatin, KRAS, Neuropathy

## Abstract

**Background:**

KRAS mutations are fraught with the progression of colorectal cancer and resistance to chemotherapy. There are pathways such as extracellular regulated protein kinase 1/2 (ERK1/2) and Akt downstream and farnesylation and geranylgeranylation upstream that are activated upon mutated KRAS. Previous studies have shown that statins, 3-hydroxy-3-methylglutaryl coenzyme A reductase inhibitors, are effective to treat KRAS mutated colorectal cancer cells. Increased doses of oxaliplatin (L-OHP), a well-known alkylating chemotherapeutic drug, causes side effects such as peripheral neuropathy due to ERK1/2 activation in spinal cords. Hence, we examined the combinatorial therapeutic efficacy of statins and L-OHP to reduce colorectal cancer cell growth and abrogate neuropathy in mice.

**Methods:**

Cell survival and confirmed apoptosis was assessed using WST-8 assay and Annexin V detection kit. Detection of phosphorylated and total proteins was analyzed the western blotting. Combined effect of simvastatin and L-OHP was examined the allograft mouse model and L-OHP-induced neuropathy was assessed using cold plate and von Frey filament test.

**Results:**

In this study, we examined the effect of combining statins with L-OHP on induction of cell death in colorectal cancer cell lines and improvement of L-OHP-induced neuropathy in vivo*.* We demonstrated that combined administration with statins and L-OHP significantly induced apoptosis and elevated the sensitivity of KRAS-mutated colorectal cancer cells to L-OHP. In addition, simvastatin suppressed KRAS prenylation, thereby enhancing antitumor effect of L-OHP through downregulation of survivin, XIAP, Bcl-xL, and Bcl-2, and upregulation of p53 and PUMA via inhibition of nuclear factor of κB (NF-κB) and Akt activation, and induction of c-Jun N-terminal kinase (JNK) activation in KRAS-mutated colorectal cancer cells. Moreover, simvastatin enhanced the antitumor effects of L-OHP and suppressed L-OHP-induced neuropathy via ERK1/2 activation in *vivo*.

**Conclusion:**

Therefore, statins may be therapeutically useful as adjuvants to L-OHP in KRAS-mutated colorectal cancer and may also be useful in the treatment of L-OHP-induced neuropathy.

**Supplementary Information:**

The online version contains supplementary material available at 10.1186/s12935-023-02884-z.

## Background

Approximately 95% of all cases of colorectal cancer are adenocarcinomas, and the most common symptoms are abnormal bowel movements and abdominal pain; however, many cases do not have any subjective symptoms, making early detection and treatment difficult. In Japan, the total number of patients with colorectal cancer has been increasing due to westernization of dietary patterns in recent years [1]. The 5-year survival rate of colorectal cancer is high (97.3%) at stage 0; however, it decreases as the stage progresses, and the prognosis is poor (17.3%) at stage IV. Surgery is the first-line remediation for colorectal cancer, but chemotherapy is also used for unresectable advanced cases, recurrent cases, or as adjuvant therapy. FOLFOX, a combination of platinum-based oxaliplatin (L-OHP), 5-fluorouracil, and levofolinate, and XELOX, a combination of L-OHP and capecitabine, are widely used as the first-line chemotherapy for colorectal cancer [2]. In addition, the first-line chemotherapy for patients with RAS wild-type colorectal cancer includes a combination of the anti-epidermal growth factor receptor (EGFR) antibodies, cetuximab and panitumumab, in addition to conventional chemotherapy [3]. However, in patients with KRAS mutations, the combination of FOLFOX and cetuximab or panitumumab has been reported to have similar or worse response rates as compared to FOLFOX alone; therefore, cetuximab and panitumumab are not recommended for patients with KRAS mutations [4]. Thus, it is important to establish an effective therapy for RAS-mutant colorectal cancer.

RAS transfers to the membrane via prenylation by farnesyl pyrophosphate (FPP) and geranylgeranyl pyrophosphate (GGPP), intermediate products of the mevalonate pathway, and transmits signals downstream by activation [5, 6]. In addition, mutations in the RAS are involved in promoting tumor growth and survival, and decreasing the sensitivity to anticancer drugs via permanent activation of the RAS protein [7]. Furthermore, RAS mutations have been identified in approximately 40% of patients with colorectal cancer [8], suggesting that RAS may be a target molecule for cancer therapy. In our previous studies, we demonstrated that statins, 3-hydroxy-3-methylglutaryl coenzyme A (HMG-CoA) reductase inhibitor, inhibit the biosynthesis of FPP and GGPP, and suppress prenylation of small G-proteins such as RAS and downstream signaling [9–11]. In addition, lovastatin enhanced the sensitivity to 5-fluorouracil and cisplatin via suppression of GGPP production in human colorectal cancer cells [12]. These results suggest that statins may be useful for treating RAS-mutant colorectal cancer.

L-OHP is the third-generation platinum-based drug designed to reduce nephrotoxicity and other toxic effects of cisplatin, and exhibits anticancer effects by inducing direct killing of the cells. In addition, L-OHP promotes T cell cytotoxicity via egress of high-mobility group box 1 protein and exsertion of calreticulin into human and murine colon cancer cells [13]. However, the occurrence of peripheral neuropathy has been reported in approximately 90% of patients treated with L-OHP, which has led to a reduction in dosage, treatment modification or discontinuation, and decreased quality of life (QOL) of patients [14]. The mechanism underlying peripheral neuropathy involves action of oxalate generated by L-OHP and biotransformation on neuronal voltage-gated sodium channels, calcium channel α2δ-1 subunit, and transient receptor potential (TRP) A1 [15–17]. It has also been suggested that L-OHP induces neuropathy by activating protein kinase C (PKC) in the central nervous system and involving the mitogen activated protein kinase (MAPK) pathway located downstream of PKC [18]. In addition, we demonstrated an increase in phosphorylated extracellular signal regulated kinase 1/2 (ERK1/2) level with activation of PKC in the fourth to sixth lumbar spinal cord in a mouse model of L-OHP-induced peripheral neuropathy; moreover, we reported the inhibitory effects of PKC inhibitors and mitogen activated protein kinase kinase (MEK) inhibitors on L-OHP-induced neuropathy [18]. Therefore, inhibition of ERK1/2 may inhibit L-OHP-induced neuropathy. Since statins have been reported to inhibit ERK1/2 [9–11], they are likely to inhibit L-OHP-induced neuropathy.

In this study, we investigated the effect of combinations of statins with L-OHP, an existing anticancer drug, on suppression of cell survival in colorectal cancer cell lines and improvement in L-OHP-induced neuropathy in vivo.

## Methods

### Cell lines and reagents

LoVo, Colo-205, Caco-2, and Colon-26 cells were obtained from the Riken Cell Bank (Ibaraki, Japan) and maintained in RPMI-1640 medium (Sigma, St Louis, MO, USA) and fetal bovine serum (FBS; Gibco, Carlsbad, CA, USA) in the presence of 5% CO_2_ at 37 °C. SW948 cells were obtained from the Japanese Collection of Research Bioresources (Osaka, Japan) and maintained in L-15 medium (Sigma) supplemented with FBS (Gibco) in the presence of 5% CO_2_ at 37 °C. HT-29 cells were purchased from DS Pharma Biomedical (Osaka, Japan) and maintained in McCoy’s 5a medium (Sigma) supplemented with FBS (Gibco) in the presence of 5% CO_2_ and temperature at 37 °C. L-OHP was purchased from LC Laboratories (Woburn, MA, USA). Simvastatin and fluvastatin were obtained from FUJIFILM Wako (Tokyo, Japan).

### Cell survival and apoptosis detection assay

Cell survival was measured by the WST-8 assay (FUJIFILM Wako) as previously described [19]. Briefly, cells were seeded in 96-well plates at 2 × 10^4^ cells/mL, and L-OHP (1–10 μM for all cell lines), simvastatin (0.5–10 μM for LoVo, Colo-205, Caco-2, SW948, and HT-29 cells, or 0.05–1 μM for Colon-26 cells), and fluvastatin (0.5–10 μM for LoVo, Colo-205, Caco-2, SW948, and HT-29 cells, or 0.05–1 μM for Colon-26 cells) were added after 1 day of pre-culture. WST-8 reagent was then added on days 1, 3, and 5, and the absorbance was measured at 490 nm using a model 680 microplate reader (Bio-Rad Laboratories, Hercules, CA, USA). Apoptosis was detected using Annexin V-FITC apoptosis detection kit (Nacalai Tesque, Inc., Kyoto, Japan) as previously described [19]. Briefly, LoVo or Colon-26 cells were added with 10 μM L-OHP, and 0.1 or 1 μM simvastatin for 2 days. After incubation, the cells were washed twice in phosphate-buffered saline and incubated with Annexin V-FITC and PI solutions. The cells were incubated at room temperature for 15 min and analyzed using a BD LSRFortessa flow cytometer (Becton Dickinson, Bedford, MA, USA).

### Western blotting

Cytoplasmic and membrane fractions were collected using ProteoExtract Subcellular Proteome Extraction Kit (Calbiochem, San Diego, CA, USA). Briefly, these fractions were loaded onto a 10% sodium dodecyl sulfate-polyacrylamide gel and transferred onto polyvinylidene fluoride membranes (GE Healthcare, Buckinghamshire, UK). After blocking with 3% skim milk, the membranes were allowed to react with following antibodies [10]: K-Ras (sc-30, RRID: AB_627865), Na + /K + -ATPase α (sc-28800, RRID: AB_2290063), anti-Bcl-2 (sc-7382, RRID: AB_626736), anti-Bcl-xL (sc-8392, RRID: AB_626739), anti-Bax (sc-7480, RRID: AB_626729), anti-Bim (sc-374358, RRID: AB_10987853), anti-p53 (sc-126, RRID: AB_628082), anti-NOXA (sc-56169, RRID: AB_784877), anti-PUMA (sc-374223, RRID: AB_10987708) (Santa Cruz Biotechnologies, CA, USA), anti-β-actin (A2228, RRID: AB_476697, Sigma), anti-phospho-Akt (#9271, RRID: AB_329825), anti-Akt (#9272, RRID: AB_329827), anti-phospho-ERK1/2 (#9101, RRID: AB_331646), anti-ERK1/2 (#9102, RRID: AB_330744), anti-phospho-c-Jun N-terminal kinase (JNK) (#9251, RRID: AB_331659), anti-JNK (#9252, RRID: AB_2250373), anti-phospho-NF-κB p65 (#3031, RRID: AB_330559), anti-NF-κB p65 (#8242, RRID: AB_10859369), anti-XIAP (#2042, RRID: AB_2214870), and anti-survivin (#2803, RRID: AB_490807) antibody (Cell Signaling Technology, Beverly, MA, USA). After reacting with secondary antibodies (horseradish peroxidase-coupled anti-rabbit (#7074) and anti-mouse (#7076); RRID: AB_2099233 and AB_330924, Cell Signaling Technology), proteins were detected using a Luminata Forte (Merck Millipore, Nottingham, UK). The amount of detected proteins were measured based on densitometry by a CS analyzer (ATTO, Tokyo, Japan) and detected proteins were standardized to the corresponding proteins [20].

### Allograft mouse model

Colon-26 cells were injected subcutaneously into the left flank of Balb/c mice (male, age 5 weeks, Shimizu Laboratory Animals, Kyoto, Japan) at 1 × 10^6^ cells in 50 μL phosphate-buffered saline (PBS). One week after inoculation (Day 0), mice were randomized and started to administer the drug in mice with subcutaneous tumors of approximately 50 mm^3^. Mice (n = 9 per group) received three intravenous injections of L-OHP (6 or 10 mg/kg) or vehicle (5% glucose solution) on days 0, 7, and 14; on day 0, simvastatin was administered 12 h after L-OHP administration. The mice were treated orally (p.o.) with 10 or 50 mg/kg simvastatin once daily for 3 weeks. Tumor size was determined daily using calipers, and tumor volume was computed using the formula (a x b^2^)/2 (a and b represent the major and minor diameters, respectively). All of the experiments were conducted in a blinded manner throughout the experiments.

### Neuropathy assay

Cold sensitivity was analyzed using a hot/cold plate (Ugo Basile, Milan, Italy). After acclimating the mice to the test apparatus for 1 h, the mice were individually placed in the center of a cold plate maintained at 10 °C. Behaviors associated with cold hypersensitivity (e.g., hind paw lifting and licking) were appreciated and the time(s) at which the first sign appeared was recorded. The cut-off time for latency of paw lifting or licking behavior was 30 s [21, 22].

Mechanical allodynia was analyzed using the von Frey filaments (Ugo Basile) with bending forces (0.16 g). Mice were placed in a 20 cm × 20 cm transparent plastic box with a metal mesh floor at a height of 20 cm from the bench and allowed to acclimate to this environment for 15 min prior to the test. Five stimuli were applied to the filament at intervals of 3 to 5 s. Mechanical sensitivity was evaluated as follows. Mechanical sensitivity was evaluated in the following order: 0: no response, 1: withdrawal of the paw, 2: immediate flinching of the stimulated paw [21, 23, 24]. The pain scores obtained from both hind paws of each mouse for the five stimuli were averaged and recorded.

Cold sensitivity and mechanical allodynia analyses were performed daily from days 0 to 21. All of the experiments were conducted in a blinded manner throughout the experiments.

All animal experiments were approved by the Animal Care and Use Committee of Kindai University (project identification code KAPS-2020-011, April 1, 2020).

### Statistical analysis

In vitro or in vivo assay results were shown as means and standard deviations (SDs) or standard error of the mean (SEMs). Statistical analysis was performed using ANOVA with Dunnett’s test using IBM SPSS version 21.0 (IBM, Armonk, NY, USA), and the difference was considered significant when *P* < 0.05.

## Results

### Effect of L-OHP, simvastatin, and fluvastatin on colorectal cancer cell survival and statins enhanced the sensitivity to L-OHP in KRAS-mutated colorectal cancer cells

**Fig. 1:**
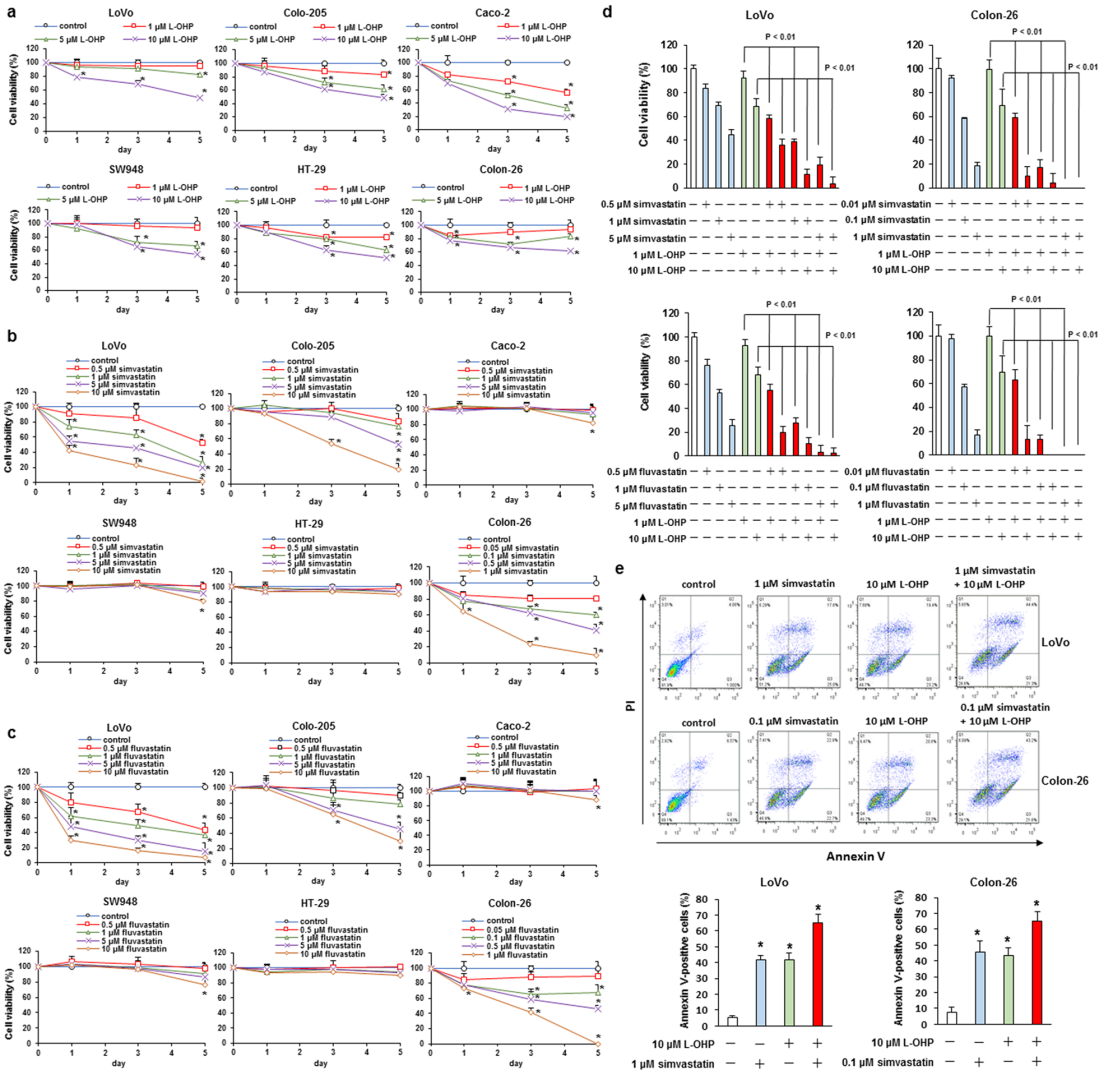
Effect of simvastatin, fluvastatin, or L-OHP alone, and combined treatment with statins and L-OHP on cell viability in KRAS-, BRAF-, or PIK3CA-mutated colorectal cancer cells. **a**–**c** LoVo, Colo-205, Caco-2, SW948, HT-29, and Colon-26 cells were administered with the indicated concentrations of **a** L-OHP, **b** simvastatin, or **c** fluvastatin for 1, 3, and 5 days. Cell survival was measured by the WST-8 assay. The results are exemplary five independent experiments. Mean values and S.D. are shown. Statistical analysis was performed by ANOVA with Dunnett’s, and the difference was considered significant when *P* < 0.05. **d** LoVo and Colon-26 cells were administered with the showed concentrations of the combined therapy of statins and L-OHP for 3 days. Cell survival was measured by the WST-8 assay. The results are exemplary five independent experiments. Mean values and S.D. are shown. Statistical analysis was performed by ANOVA with Dunnett’s, and the difference was considered significant when *P* < 0.05. **e** LoVo and Colon-26 cells were administered with the showed concentrations of the combined therapy of simvastatin and L-OHP for 2 days. Apoptosis was detected using Annexin V-FITC apoptosis detection kit. Mean values and S.D. are shown. The results are exemplary five independent experiments. Statistical analysis was performed by ANOVA with Dunnett’s, and the difference was considered significant when *P* < 0.05

We explored the cytotoxic effect of L-OHP, simvastatin, and fluvastatin in LoVo (KRAS G13D mutation), Colo-205 (BRAF V600E mutation), Caco-2 (KRAS, BRAF, and PIK3CA wild-type), SW948 (PIK3CA E542K mutation), HT-29 (BRAF V600E and PIK3CA P449T mutation), and Colon-26 (KRAS G12D mutation) cells. These cells were administered with L-OHP (1–10 μM), simvastatin (0.5–10 μM or 0.05–1 μM), or fluvastatin (0.5–10 μM or 0.05–1 μM), and cell viability was investigated. Treatment with L-OHP markedly declined the cell viability in Caco-2 cells, but other cell lines (LoVo, Colo-205, SW948, HT-29, and Colon-26 cells) showed weakly-induced cell death (Fig. 1a and Additional file 1: Fig. S1). In addition, administration with simvastatin and fluvastatin remarkably induced cell death in LoVo and Colon-26 cells, moderately promoted cell death in Colo-205 cells, and did not affect cell viability in Caco-2, SW948, and HT-29 cells (Fig. 1b, c and Additional file 1: Fig. S1). Next, we explored the effect of combined administration of statins and L-OHP in LoVo and Colon-26 cells. Treatment with 0.5–5 μM simvastatin or fluvastatin elevated the response rate of 1 or 10 μM L-OHP in LoVo and Colon-26 cells, and the combination showed synergistic effect in apoptosis induction (Fig. 1d, e and Additional file 1: Fig. S2). These results indicate that statins augment the anticancer effects of L-OHP in colorectal cancer cells.

### Statins suppressed the Akt and NF-κB p65 activation via inhibition of Ras membrane localization in KRAS mutated colorectal cancer cells

**Fig. 2:**
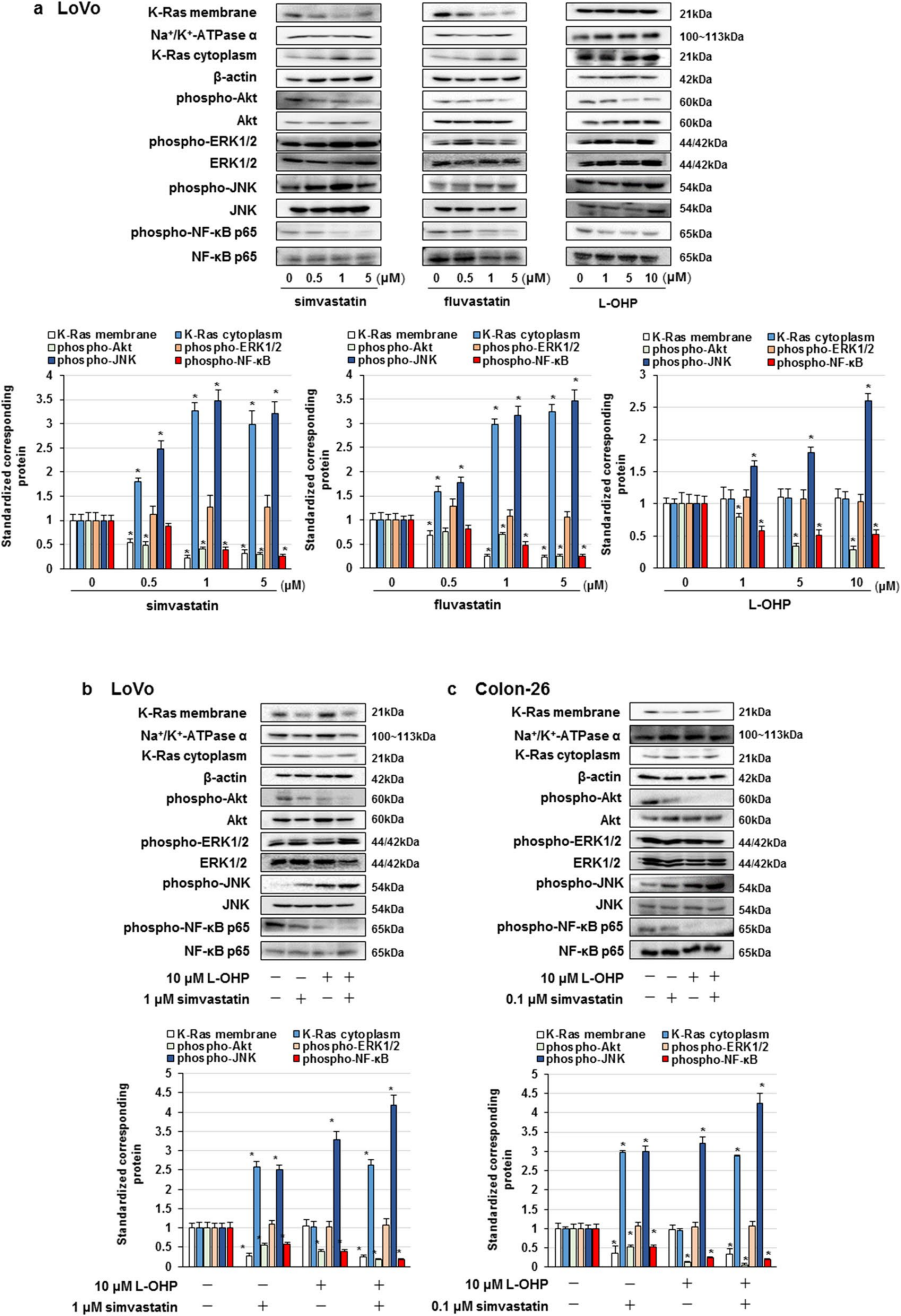
Effect of statins and L-OHP on Ras localization, Akt, ERK1/2, JNK, and NF-κB activation. **a**–**c** Cells were administered with statins or L-OHP. **a** LoVo cells were treated with statins or L-OHP alone for 3 days. **b**, **c** LoVo and Colon-26 cells were co-treated with simvastatin and L-OHP for 3 days. Protein detected by western blot assay and the amount of detected proteins were measured based on densitometry. The results are exemplary four independent experiments. Mean values and S.D. are shown. Statistical analysis was performed by ANOVA with Dunnett’s, and the difference was considered significant when *P* < 0.05

Statins promoted cell death via GGPP production, an intermediate factor of the mevalonate pathway, and inhibition of small GTPase prenylation [10]. We examined the effect of administration of simvastatin, fluvastatin, or L-OHP alone on the representative signaling pathways that mediate cell survival, including Akt, ERK1/2, JNK, and NF-κB p65, in LoVo cells. Simvastatin and fluvastatin inhibited Akt and NF-κB p65 phosphorylation and increased the phosphorylation of JNK via suppression of K-Ras membrane localization, but did not affect the ERK1/2 phosphorylation in LoVo cells (Fig. 2a). In addition, L-OHP suppressed Akt and NF-κB activation and induced JNK activation in LoVo cells (Fig. 2a). Moreover, combination of 1 or 0.1 μM simvastatin and 10 μM L-OHP markedly inhibited Akt and NF-κB p65 phosphorylation and promoted JNK phosphorylation in LoVo and Colon-26 cells (Fig. 2b, c). These results suggest that co-treatment with statins and L-OHP abrogate Akt and NF-κB activation, and promote JNK activation in LoVo and Colon-26 cells.

### Combined treatment simvastatin and L-OHP suppressed the XIAP, Bcl-xL, and Bcl-2 expression and elevated expression of p53 and PUMA

**Fig. 3:**
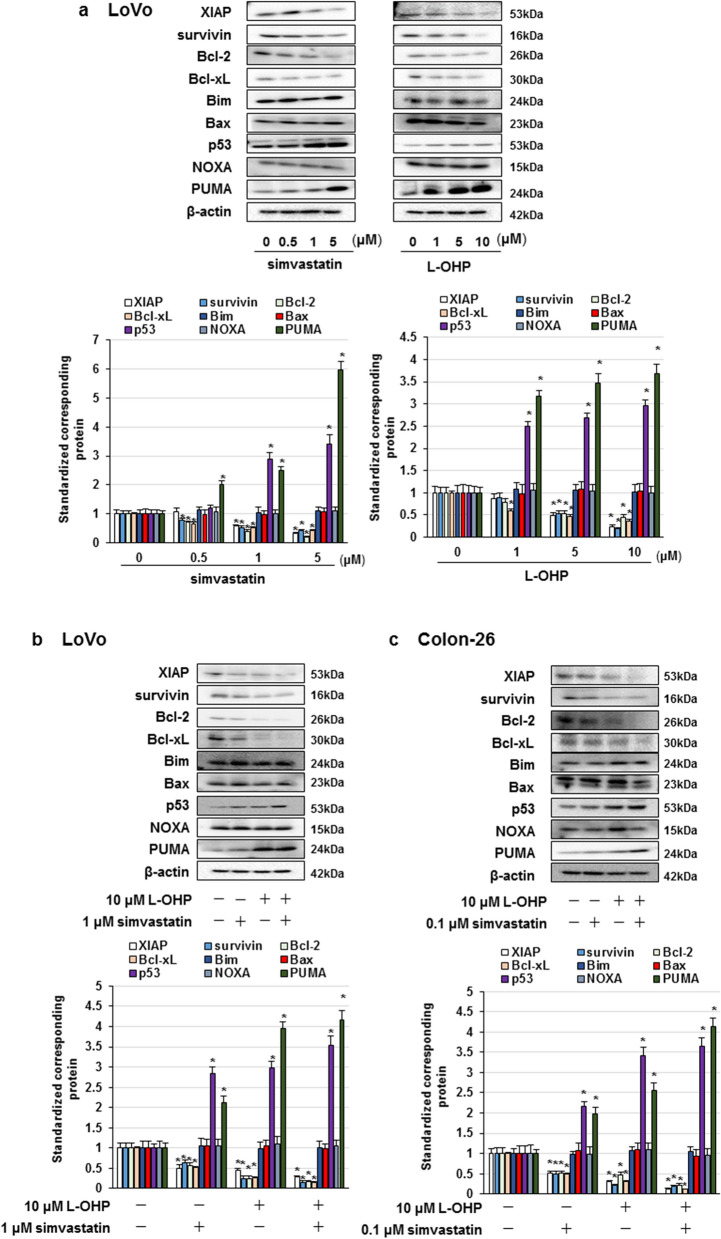
Effect of statins and L-OHP on XIAP, survivin, Bcl-2, Bcl-xL, Bim, Bax, p53, NOXA, and PUMA expression. **a**–**c** Cells were administered with statins or L-OHP. **a** LoVo cells were treated with statins or L-OHP alone for 3 days. **b**, **c** LoVo and Colon-26 cells were co-treated with simvastatin and L-OHP for 3 days. Protein detected by western blot assay and the amount of detected proteins were measured based on densitometry. The results are exemplary four independent experiments. Mean values and S.D. are shown. Statistical analysis was performed by ANOVA with Dunnett’s, and the difference was considered significant when *P* < 0.05

Considering that Akt, NF-κB p65, and JNK regulate the expression of pro-apoptotic and anti-apoptotic factors [19, 25], we explored the effect of simvastatin and L-OHP on the expression of pro-apoptotic factors, such as Bim, Bax, p53, NOXA, and PUMA, and anti-apoptotic factors, such as survivin, XIAP, Bcl-xL, and Bcl-2. We found that simvastatin or L-OHP alone downregulated survivin, XIAP, Bcl-xL, and Bcl-2 expression, and upregulated the expression levels of p53 and PUMA in LoVo cells (Fig. 3a). In addition, co-treatment with simvastatin and L-OHP conspicuously inhibited survivin, XIAP, Bcl-xL, and Bcl-2 expression, and augmented p53 and PUMA expression in LoVo and Colon-26 cells (Fig. 3b, c). These results indicate that co-administration of statins and L-OHP abrogate the expression of survivin, XIAP, Bcl-xL, and Bcl-2, and enhance p53 and PUMA expression through the suppression of Akt and NF-κB activation and promotion of JNK activation, thereby inducing apoptosis in LoVo and Colon-26 cells.

### Simvastatin enhanced the tumor growth-inhibiting efficacy of L-OHP

**Fig. 4:**
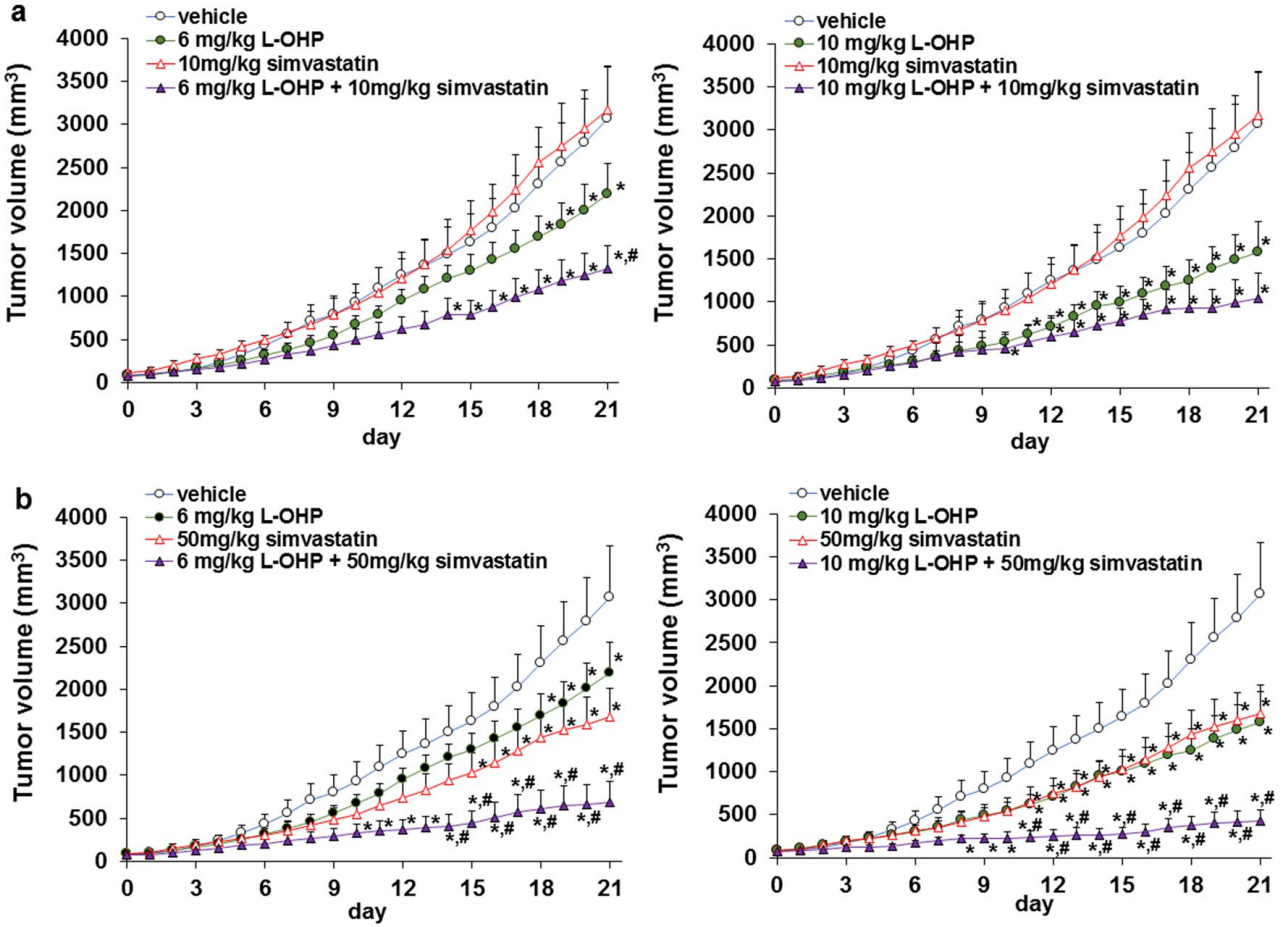
Effect of simvastatin and L-OHP on Colon-26 tumor growth. **a** Treatment with 6 or 10 mg/kg L-OHP, and 10 mg/kg simvastatin for 3 weeks. **b** Treatment with 6 or 10 mg/kg L-OHP, and 50 mg/kg simvastatin for 3 weeks. Mean values and S.E.M. are shown. Statistical analysis was performed by ANOVA with Dunnett’s, and the difference was considered significant when *P* < 0.05. *P < 0.05; versus vehicle and #*P* < 0.05; versus 6 or 10 mg/kg oxaliplatin

It is important to confirm the combined effects of the above statins and L-OHP in vivo. Next, we explored the effect of simvastatin on antitumor activity of L-OHP in vivo. Treatment with 50 mg/kg simvastatin alone (p.o.) suppressed tumor growth, whereas 10 mg/kg simvastatin alone did not. In addition, 6 or 10 mg/kg L-OHP alone (i.v.) inhibited tumor growth. Moreover, the combination of 50 mg/kg simvastatin and 6 or 10 mg/kg L-OHP significantly inhibited tumor growth compared to 6 or 10 mg/kg L-OHP alone without weight loss in mice (Fig. 4a, b and Additional file 1: Fig. S3). These results indicate that statins augmented the antitumor effect of L-OHP in vivo.

### Simvastatin suppressed the L-OHP-induced neuropathy

**Fig. 5:**
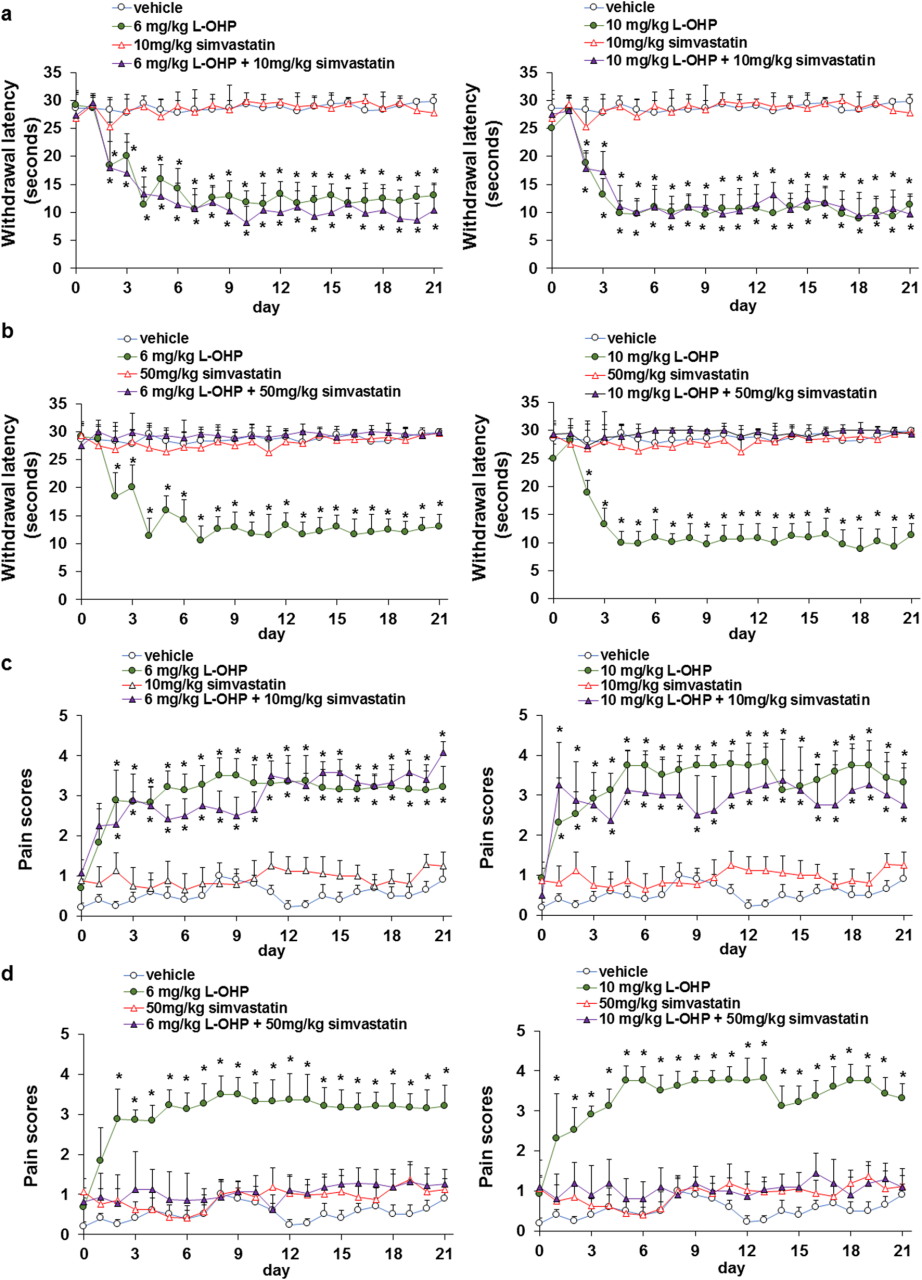
Simvastatin suppressed LOHP-induced neuropathy. **a**, **b** Cold sensitivity was analyzed using a hot/cold plate. Mean values and S.E.M. are shown. Statistical analysis was performed by ANOVA with Dunnett’s, and the difference was considered significant when P < 0.05. **c**, **d** Mechanical allodynia was analyzed using the von Frey filaments (Ugo Basile) with bending forces (0.16 g). Mean values and S.E.M. are shown. Statistical analysis was performed by ANOVA with Dunnett’s, and the difference was considered significant when *P* < 0.05

L-OHP is a key drug present in FOLFOX and XELOX, the first-line therapies for colorectal cancer; however, it frequently induces neuropathy, resulting in discontinuation of treatment or reduction of dosage, which leads to decreased QOL of patients. Using the cold plate and von Frey filament tests, we investigated whether simvastatin suppressed L-OHP-induced cold and mechanical allodynia. Combination treatment with 50 mg/kg simvastatin, but not with 10 mg/kg simvastatin, suppressed L-OHP-induced cold and mechanical allodynia via suppression of ERK1/2 phosphorylation in the lumbar spinal cord of mice (Fig. 5, Additional file 1: Fig. S4-S6, and Additional file 2). These results suggest that statins not only potentiate the antitumor effects of L-OHP, but also attenuate L-OHP-induced neuropathy.

## Discussion

In this study, we demonstrated that KRAS-mutant colorectal cancers were less sensitive to L-OHP and more sensitive to statins, and statins improved L-OHP hyposensitivity and cytotoxic effect in KRAS-mutated colorectal cancer cells. It has been reported that KRAS mutations in patients with colorectal cancer receiving FOLFOX-4 therapy were involved with shorter overall survival (OS) and disease-free survival (DFS) [26]. Additionally, simvastatin resensitized patients with cetuximab-resistant KRAS-mutated colorectal cancer to cetuximab, and taking a statin after colorectal cancer diagnosis reduced the risk of death from colorectal cancer [27, 28]. Recently, sotorasib was approved by the FDA for KRAS G12C mutation in non-small cell lung cancer, but drugs for the treatment (Additional file 2) of other KRAS mutations are still not available. In addition, acquired resistance to sotorasib and adagrasib, KRAS G12C mutation inhibitors, was associated with KRAS secondary mutations, such as KRAS Y96D, Y96S, G13D, and A59S [29]. In the present study, statins significantly induced cell death and enhanced the efficacy of L-OHP in KRAS G13D or G12D mutations. These results indicate that statins may be effective against KRAS mutations, including G13D, G12D, and other mutations.

Combined treatment with L-OHP and statins markedly inhibited the activation of NF-κB and Akt, expression of survivin, XIAP, Bcl-xL, and Bcl-2, and elevated JNK activation, expression of p53 and PUMA in KRAS-mutated colorectal cancer cells. In addition, statins abrogated K-Ras membrane localization in KRAS-mutated colorectal cancer cells but not in L-OHP. GGPP, an intermediate product of the mevalonate pathway, plays an important role in the membrane localization of Ras. Statins inhibit the membrane localization of Ras by abrogating the production of GGPP, resulting in increased cytoplasmic localization of Ras [10, 30]. In addition, statins have been reported to suppress the activation of small GTPases such as Ras, thereby exerting an antitumor effect [9–11]. The activation of Ras through the overexpression of miR-188-5p reduces sensitivity to L-OHP in human colorectal cancer cells [31]. Our previous study indicated that L-OHP promoted cell death via suppression of NF-κB and Akt activation, and induction of JNK activation in colorectal cancer [19]. Activation of Akt and NF-κB p65 regulated the survivin, XIAP, Bcl-xL, and Bcl-2 expression in human colorectal cancer cells [19]. In addition, perifosine inhibited the Akt pathway and enhanced p53 and PUMA expression, thereby inducing apoptosis in colorectal cancer cells [19]. Moreover, activation of JNK by CJK-7 enhanced p53 and PUMA expression and decreased the expression of Bcl-2 in HCT-116, a human colorectal cancer cell line [25]. It has also indicated that simvastatin inhibited the Bcl-xL and Bcl-2 expression via abrogated NF-κB activation in COLO205 cells [32]. These findings indicate that statins exert antitumor effect by inhibiting Ras, Akt, and NF-κB activation and inducing JNK activation, thus increasing antitumor activity of L-OHP.

L-OHP induces acute peripheral neuropathy characterized by cold-sensitive paresthesia and dysesthesia of the hand and foot in about 90% of patients (evaluated as cold allodynia in animal models) and chronic peripheral neuropathy characterized by temperature-independent paresthesia and dysesthesias of the hand and foot in about 70% of patients (evaluated as mechanical allodynia in animal models) [14, 33, 34]. These adverse events lead to dose reduction and the discontinuation of the drug, even if L-OHP continues to be effective and a decrease in the patient's QOL [14, 33]. L-OHP-induced acute peripheral neuropathy is associated with calcium channels, such as TRPM8 and TRPA1, and sodium channels in the dorsal root ganglion (DRG), whereas chronic peripheral neuropathy is correlated with damage to nerve and ganglion cells by L-OHP accumulation [34]. In addition, L-OHP-induced acute (cold allodynia) and chronic neuropathy (mechanical allodynia) are related to ERK1/2 activation in the spinal cord and DRG of mice and rats [35, 36]. We found that statins suppressed the L-OHP-induced neuropathy via inhibition of ERK1/2 phosphorylation in vivo. Previous studies have shown that ERK1/2 activation in the dorsal root ganglia and spinal cord is involved in L-OHP-induced neuropathy in mice [37]. ERK1/2 activation in the spinal cord is an indicator of chemotherapy-induced peripheral neuropathy and various types of pain, such as neuropathic pain and visceral pain [38]. These findings suggest that statins may be effective in the treatment of colorectal cancer, not only in enhancing antitumor effects, but also in controlling side effects.

In this study, we demonstrated that simvastatin (0.01–5 μM) and fluvastatin (0.01–5 μM) elevated the sensitivity of colorectal cancer cells to L-OHP in vitro, and treatment with 10 or 50 mg/kg simvastatin (p.o.) resulted in enhanced antitumor effect of L-OHP in vivo; moreover, treatment with 50 mg/kg simvastatin inhibited L-OHP -induced neuropathy in mice in vivo. It has reported that high-dose simvastatin (15 mg/kg/day) administered immediately before chemotherapy was safe and well tolerated in patients with relapsed or refractory myeloma or lymphoma; because mice metabolize statins more rapidly than humans, the 15 mg/kg/day dose in humans is equivalent to the 202 mg/kg/day dose of simvastatin used in the Hodgkin lymphoma mouse model [39]. In addition, multiple myeloma patients treated with 15 mg/kg/day of simvastatin for 7 days achieved a plasma concentration of 2.7 μM [40]. Moreover, hypercholesterolemia patients treated with 20–80 mg of fluvastatin achieved a plasma concentration of 1 μM [41]. In our study, doses of close to plasma concentrations were administered in vitro, and doses lower than those used in other cancer clinical trials were administered in vivo. These findings suggest that co-treatment with simvastatin produces beneficial effects in colorectal cancer.

Limitations of this study include that it does not confirm whether statins have an antitumor effect or L-OHP-induced peripheral neuropathy in clinical practice in patients with KRAS-mutant colorectal cancer. It has indicated that statins during and after adjuvant chemotherapy in patients with colorectal cancer have been shown not to improve DFS, recurrence-free survival, and OS in KRAS mutant and wild-type patients [42]. It has also reported that statins use did not improve progression free survival and OS in KRAS-mutated colorectal cancer patients treated with XELOX + bevacizumab with/without cetuximab [43]. However, colorectal cancer patients taking statins have been shown to have improved postoperative cancer specific 5-year survival rates compared to colorectal cancer patients not taking statins [44]. In addition, colorectal cancer patients who were taking statins prior to neoadjuvant chemotherapy had significantly lower median American Joint Committee on Cancer (AJCC) grades compared to those not taking statins, indicating that patients with lower AJCC grades had significantly better OS, DFS, cancer-specific mortality and local recurrence [45]. Moreover, the addition of simvastatin to mFOLFOX6 + cetuximab was shown to increase the efficacy of FOLFOX6 + cetuximab, reduce the incidence of side effects, including peripheral neuropathy, and prolong OS of KRAS-mutated colorectal cancer patients [46]. These observations indicate that statins may improve the prognosis and QOL of colorectal cancer patients treated with neoadjuvant or adjuvant chemotherapy and surgery; however, there are negative observations that need to be validated in future studies.

## Conclusion

We present preclinical efficacy data of statins; statins effectively enhanced antitumor effect of L-OHP in KRAS-mutated colorectal cancer and suppressed L-OHP-induced neuropathy. Therefore, statins may be therapeutically useful as adjuvants to L-OHP in KRAS-mutated colorectal cancer and may also be useful in the treatment of L-OHP-induced neuropathy.

## Supplementary Information

**Additional file 1: Fig. S1. **IC50 values of L-OHP, simvastatin, or fluvastatin in colorectal cancer cell lines. Cell survival was detected by WST-8 assy. IC50 values were computed by using the survival rate data to a logistic curve. **Fig. S2. **Combination index values of L-OHP and simvastatin or fluvastatin in LoVo and Colon26 cells. Cell survival was detected by WST-8 assy. IC50 values were computed by using the survival rate data to a logistic curve. **Fig. S3. **Safety of combined treatment with L-OHP and simvastatin in mice. Male Balb/c mice (n=9 per group) were randomized and received three intravenous injections of L-OHP (6 or 10 mg/kg) or vehicle (5% glucose solution) on days 0, 7, and 14; on day 0, simvastatin was administered 12 h after L-OHP administration. Simvastatin was treated orally (p.o.) at **a **10 or **b **50 mg/kg once a day for 3 weeks. Mice were weighed before the first treatment and every day during the treatment period. Mean values and S.E.M. are shown. **Fig. S4. **Simvastatin suppressed LOHP-induced mechanical sensitivity in mice. Male Balb/c mice (n=9 per group) were randomized and received three intravenous injections of L-OHP (6 or 10 mg/kg) or vehicle (5% glucose solution) over 3 weeks (Days 0, 7, and 14). On day 0, simvastatin was administered 12 h after L-OHP administration. Simvastatin was treated orally (p.o.) at **a **10 or **b **50 mg/kg once a day for 3 weeks. Mechanical allodynia was analyzed using the 0.4 g von Frey filaments (Ugo Basile). Pain scores obtained from both hind paws of each mouse for five stimuli were averaged and recorded. Mean values and S.E.M. are shown. Statistical analysis was performed by ANOVA with Dunnett’s, and the difference was considered significant when P < 0.05. **Fig. S5. **Simvastatin suppressed LOHP-induced mechanical sensitivity in mice. Male Balb/c mice (n=9 per group) were randomized and received three intravenous injections of L-OHP (6 or 10 mg/kg) or vehicle (5% glucose solution) over 3 weeks (Days 0, 7, and 14). On day 0, simvastatin was administered 12 h after L-OHP administration. Simvastatin was treated orally (p.o.) at **a **10 or **b **50 mg/kg once a day for 3 weeks. Mechanical allodynia was analyzed using the 1.4 g von Frey filaments (Ugo Basile). Pain scores obtained from both hind paws of each mouse for five stimuli were averaged and recorded. Mean values and S.E.M. are shown. Statistical analysis was performed by ANOVA with Dunnett’s, and the difference was considered significant when P < 0.05. **Fig. S6. **Simvastatin suppressed LOHP-induced ERK1/2 phosphorylation in the lumber spinal cord of mice. Male Balb/c mice (n=5 per group) were randomized and received three intravenous injections of L-OHP (6 or 10 mg/kg) or vehicle (5% glucose solution) over 3 weeks (Days 0, 7, and 14). On day 0, simvastatin was administered 12 h after L-OHP administration. Simvastatin was treated orally (p.o.) at 10 or 50 mg/kg once a day for 3 weeks. After 3 weeks, the lumbar spinal cords of mice were quickly dissected and homogenized, and analyzed by western blot using the anti-phospho-ERK1/2 and anti-ERK1/2 antibodies. The amount of detected proteins were measured based on densitometry. The results are exemplary five independent experiments. Mean values and S.D. are shown. Statistical analysis was performed by ANOVA with Dunnett’s, and the difference was considered significant when P < 0.05.

**Additional File 2:** Supplementary materials and methods.

## Data Availability

The datasets used and/or analyzed during the current study are available from the corresponding author on reasonable request.
