# Supplementary material for: Statins enhances antitumor effect of oxaliplatin in KRAS-mutated colorectal cancer cells and inhibits oxaliplatin-induced neuropathy
Source: Cancer Cell Int. 2023 Apr 17;23:73. doi: 10.1186/s12935-023-02884-z (PMC10108455; doi:10.1186/s12935-023-02884-z)
Supplement: Supplementary file 1 — Additional file 1: Fig. S1. IC50 values of L-OHP, simvastatin, or fluvastatin in colorectal cancer cell lines. Cell survival was detected by WST-8 assy. IC50 values were computed by using the survival rate data to a logistic curve. Fig. S2. Combination index values of L-OHP and simvastatin or fluvastatin in LoVo and Colon26 cells. Cell survival was detected by WST-8 assy. IC50 values were computed by using the survival rate data to a logistic curve. Fig. S3. Safety of combined treatment with L-OHP and simvastatin in mice. Male Balb/c mice (n=9 per group) were randomized and received three intravenous injections of L-OHP (6 or 10 mg/kg) or vehicle (5% glucose solution) on days 0, 7, and 14; on day 0, simvastatin was administered 12 h after L-OHP administration. Simvastatin was treated orally (p.o.) at a 10 or b 50 mg/kg once a day for 3 weeks. Mice were weighed before the first treatment and every day during the treatment period. Mean values and S.E.M. are shown. Fig. S4. Simvastatin suppressed LOHP-induced mechanical sensitivity in mice. Male Balb/c mice (n=9 per group) were randomized and received three intravenous injections of L-OHP (6 or 10 mg/kg) or vehicle (5% glucose solution) over 3 weeks (Days 0, 7, and 14). On day 0, simvastatin was administered 12 h after L-OHP administration. Simvastatin was treated orally (p.o.) at a 10 or b 50 mg/kg once a day for 3 weeks. Mechanical allodynia was analyzed using the 0.4 g von Frey filaments (Ugo Basile). Pain scores obtained from both hind paws of each mouse for five stimuli were averaged and recorded. Mean values and S.E.M. are shown. Statistical analysis was performed by ANOVA with Dunnett’s, and the difference was considered significant when P < 0.05. Fig. S5. Simvastatin suppressed LOHP-induced mechanical sensitivity in mice. Male Balb/c mice (n=9 per group) were randomized and received three intravenous injections of L-OHP (6 or 10 mg/kg) or vehicle (5% glucose solution) over 3 weeks (Days 0, 7, and 14). On day 0, [file 12935_2023_2884_MOESM1_ESM.pdf]

Cell line	IC_{50} (μM)		
	L-OHP	simvastatin	fluvastatin
LoVo	9.389	0.500	0.430
Colo205	9.083	4.132	4.636
Caco-2	1.512	> 10	> 10
SW948	> 10	> 10	> 10
HT29	> 10	> 10	> 10
Colon-26	> 10	0.238	0.256

Fig. S1. IC50 values of L-OHP, simvastatin, or fluvastatin in colorectal cancer cell lines. Cell survival was detected by WST-8 assy. IC50 values were computed by using the survival rate data to a logistic curve.

Cell line	conbination	CI value
LoVo	0.5 μM simvastatin + 1 μM L-OHP	0.565
	0.5 μM simvastatin + 10 μM L-OHP	0.877
	1 μM simvastatin + 1 μM L-OHP	0.372
	1 μM simvastatin + 10 μM L-OHP	0.312
	5 μM simvastatin + 1 μM L-OHP	0.657
	5 μM simvastatin + 10 μM L-OHP	0.502
	0.5 μM fluvastatin + 1 μM L-OHP	0.689
	0.5 μM fluvastatin + 10 μM L-OHP	0.418
	1 μM fluvastatin + 1 μM L-OHP	0.309
	1 μM fluvastatin + 10 μM L-OHP	0.329
	5 μM fluvastatin + 1 μM L-OHP	0.425
	5 μM fluvastatin + 10 μM L-OHP	0.572
Colon-26	0.01 μM simvastatin + 1 μM L-OHP	0.386
	0.01 μM simvastatin + 10 μM L-OHP	0.443
	0.1 μM simvastatin + 1 μM L-OHP	0.101
	0.1 μM simvastatin + 10 μM L-OHP	0.388
	1 μM simvastatin + 1 μM L-OHP	0.150
	1 μM simvastatin + 10 μM L-OHP	0.463
	0.01 μM fluvastatin + 1 μM L-OHP	0.525
	0.01 μM fluvastatin + 10 μM L-OHP	0.503
	0.1 μM fluvastatin + 1 μM L-OHP	0.081
	0.1 μM fluvastatin + 10 μM L-OHP	0.326
	1 μM fluvastatin + 1 μM L-OHP	0.141
	1 μM fluvastatin + 10 μM L-OHP	0.452

Fig. S2. Combination index values of L-OHP and simvastatin or fluvastatin in LoVo and Colon26 cells. Cell survival was detected by WST-8 assy. IC50 values were computed by using the survival rate data to a logistic curve.

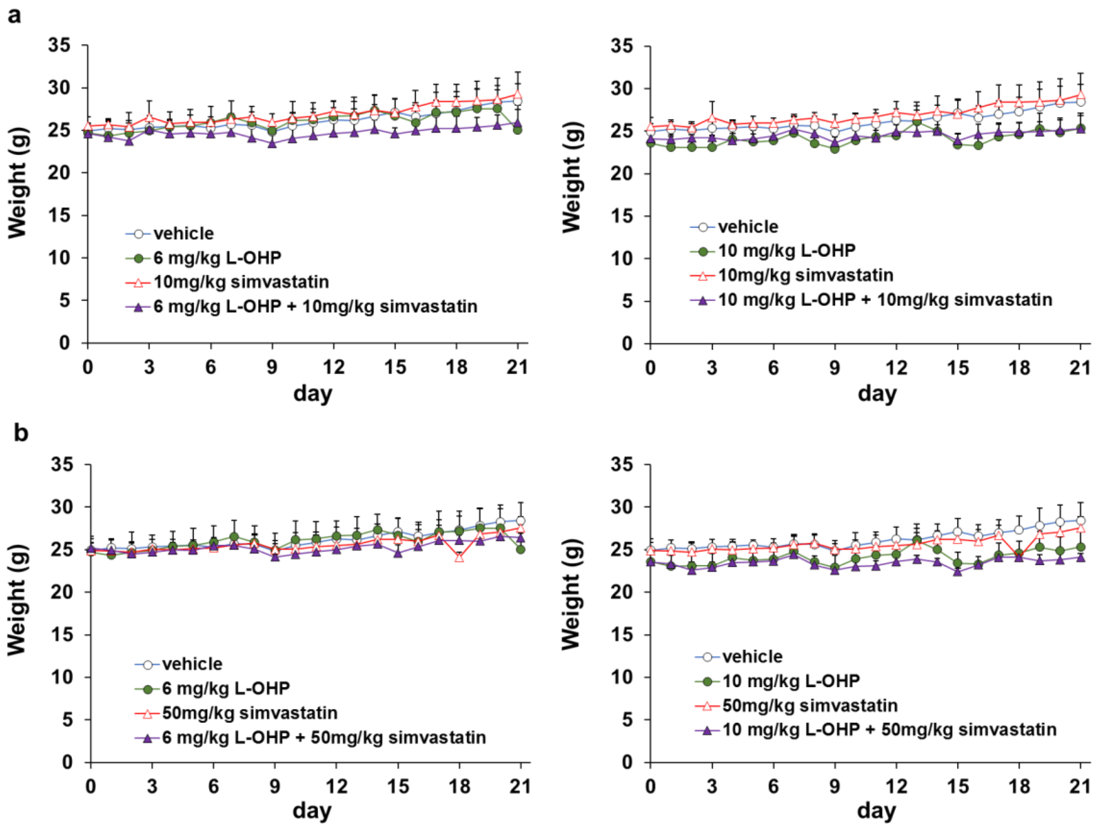

Fig. S3. Safety of combined treatment with L-OHP and simvastatin in mice. Male Balb/c mice (n=9 per group) were randomized and received three intravenous injections of L-OHP (6 or 10 mg/kg) or vehicle (5% glucose solution) on days 0, 7, and 14; on day 0, simvastatin was administered 12 h after L-OHP administration. Simvastatin was treated orally (p.o.) at **a** 10 or **b** 50 mg/kg once a day for 3 weeks. Mice were weighed before the first treatment and every day during the treatment period. Mean values and S.E.M. are shown.

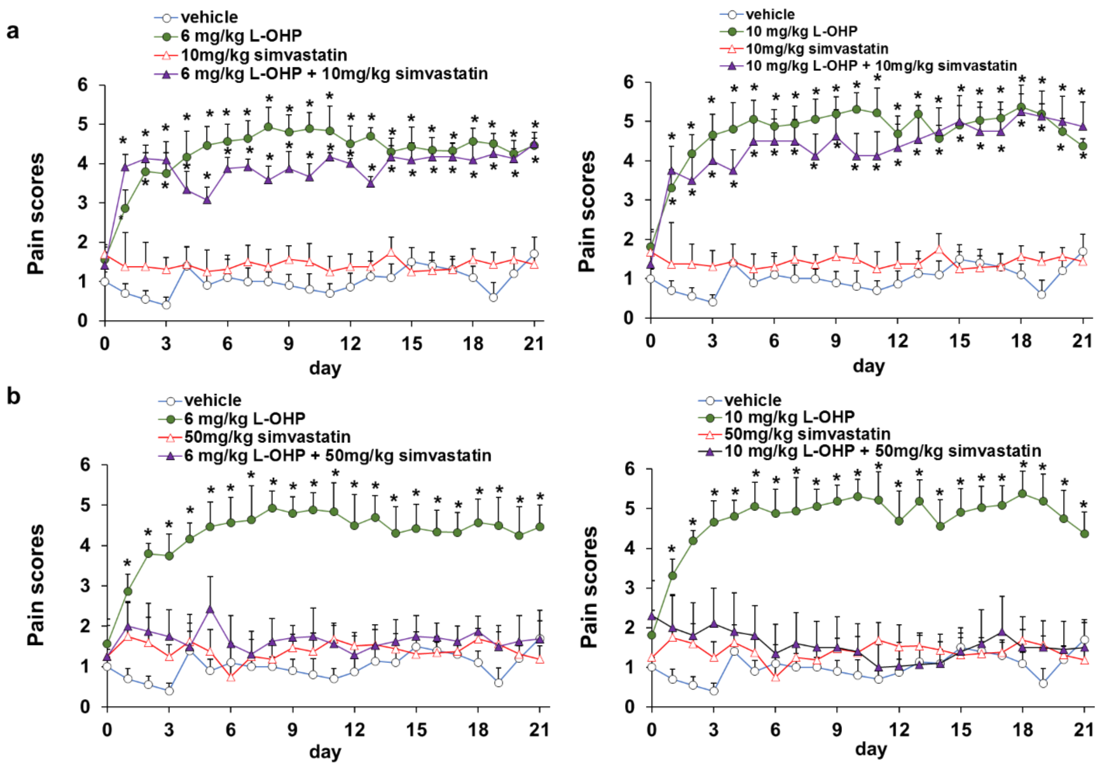

Fig. S4. Simvastatin suppressed LOHP-induced mechanical sensitivity in mice. Male Balb/c mice (n=9 per group) were randomized and received three intravenous injections of L-OHP (6 or 10 mg/kg) or vehicle (5% glucose solution) over 3 weeks (Days 0, 7, and 14). On day 0, simvastatin was administered 12 h after L-OHP administration. Simvastatin was treated orally (p.o.) at **a** 10 or **b** 50 mg/kg once a day for 3 weeks. Mechanical allodynia was analyzed using the 0.4 g von Frey filaments (Ugo Basile). Pain scores obtained from both hind paws of each mouse for five stimuli were averaged and recorded. Mean values and S.E.M. are shown. Statistical analysis was performed by ANOVA with Dunnett's, and the difference was considered significant when $P < 0.05$.

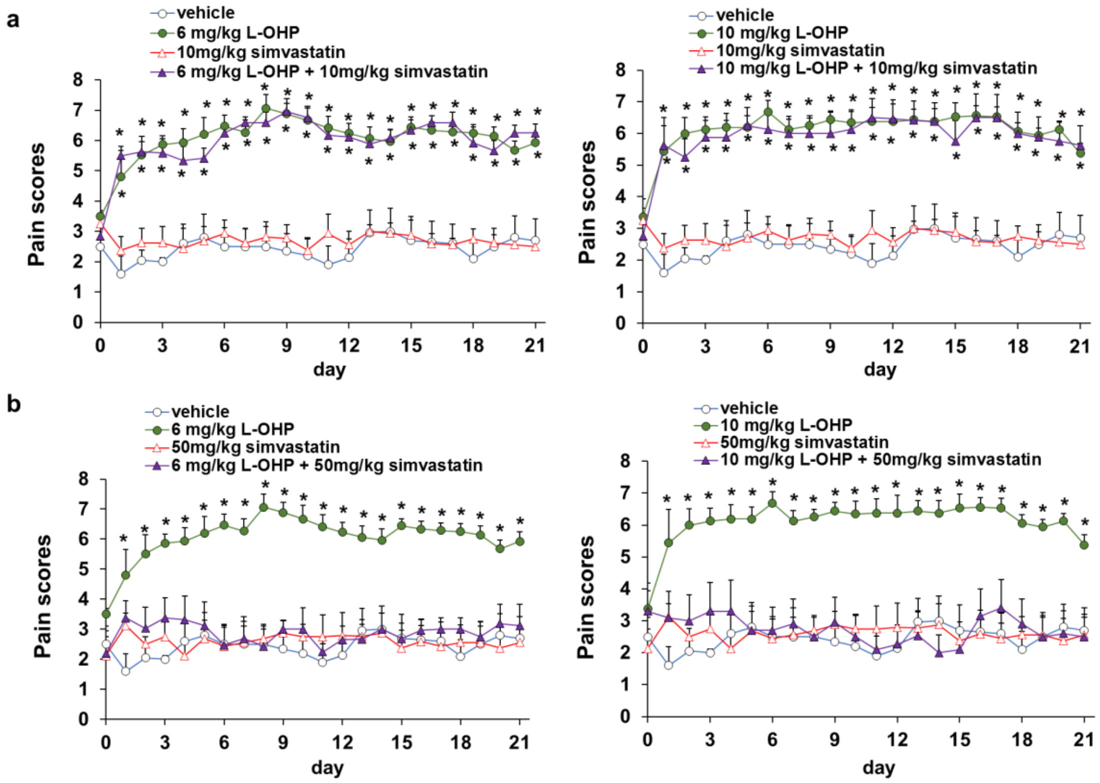

Fig. S5. Simvastatin suppressed LOHP-induced mechanical sensitivity in mice. Male Balb/c mice (n=9 per group) were randomized and received three intravenous injections of L-OHP (6 or 10 mg/kg) or vehicle (5% glucose solution) over 3 weeks (Days 0, 7, and 14). On day 0, simvastatin was administered 12 h after L-OHP administration. Simvastatin was treated orally (p.o.) at **a** 10 or **b** 50 mg/kg once a day for 3 weeks. Mechanical allodynia was analyzed using the 1.4 g von Frey filaments (Ugo Basile). Pain scores obtained from both hind paws of each mouse for five stimuli were averaged and recorded. Mean values and S.E.M. are shown. Statistical analysis was performed by ANOVA with Dunnett’s, and the difference was considered significant when $P < 0.05$.

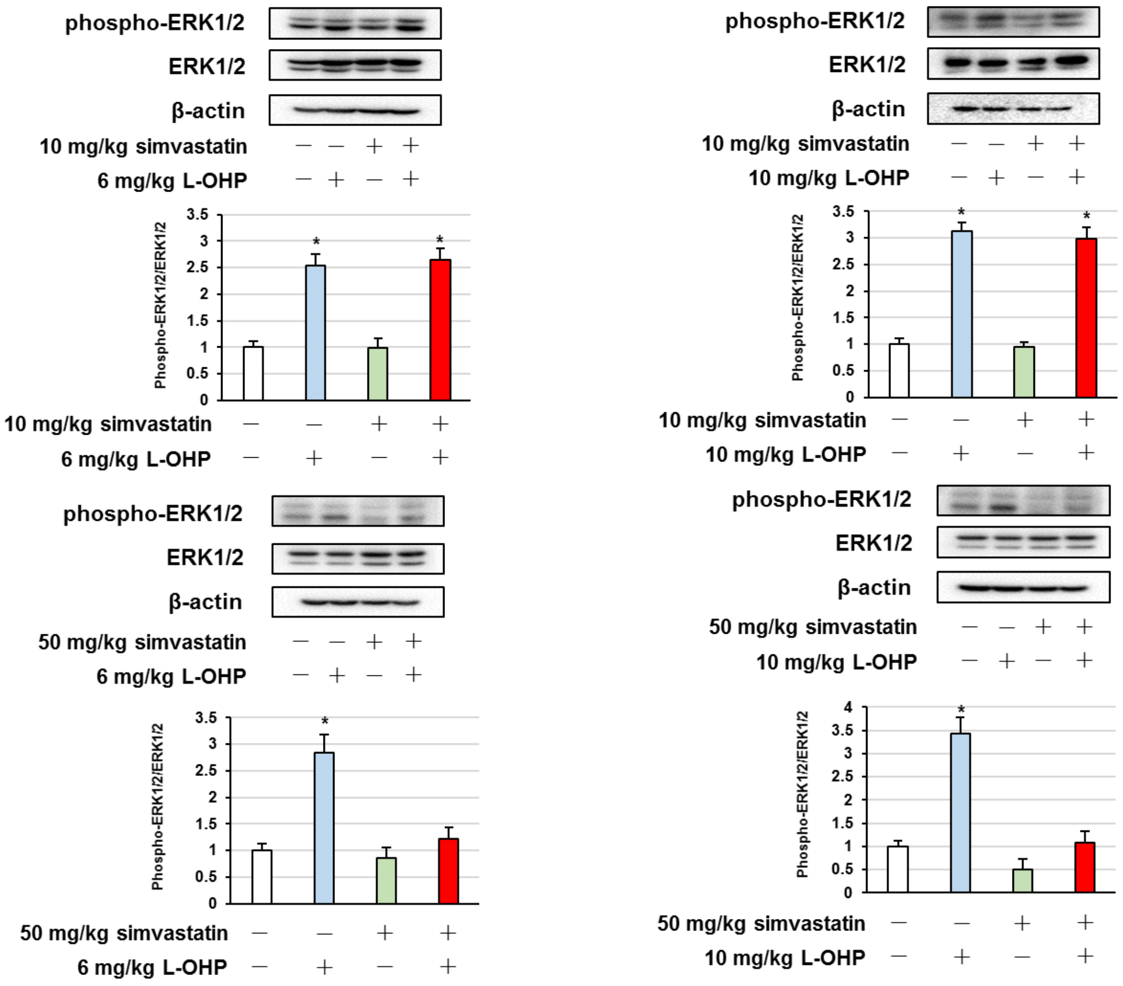

Fig. S6. Simvastatin suppressed LOHP-induced ERK1/2 phosphorylation in the lumber spinal cord of mice. Male Balb/c mice (n=5 per group) were randomized and received three intravenous injections of L-OHP (6 or 10 mg/kg) or vehicle (5% glucose solution) over 3 weeks (Days 0, 7, and 14). On day 0, simvastatin was administered 12 h after L-OHP administration. Simvastatin was treated orally (p.o.) at 10 or 50 mg/kg once a day for 3 weeks. After 3 weeks, the lumbar spinal cords of mice were quickly dissected and homogenized, and analyzed by western blot using the anti-phospho-ERK1/2 and anti-ERK1/2 antibodies. The amount of detected proteins were measured based on densitometry. The results are exemplary five independent experiments. Mean values and S.D. are shown. Statistical analysis was performed by ANOVA with Dunnett's, and the difference was considered significant when $P < 0.05$.
