# Supplementary material for: Statins enhances antitumor effect of oxaliplatin in KRAS-mutated colorectal cancer cells and inhibits oxaliplatin-induced neuropathy
Source: Cancer Cell Int. 2023 Apr 17;23:73. doi: 10.1186/s12935-023-02884-z (PMC10108455; doi:10.1186/s12935-023-02884-z)
Supplement: Supplementary file 2 — Additional File 2: Supplementary materials and methods. [file 12935_2023_2884_MOESM2_ESM.pdf]

Supplementary materials and methods

Analysis of ERK1/2 activation in mouse lumbar spinal cord

After the mice were euthanized with CO_2, the lumbar spinal cords (lumbar segments 4–6) were quickly dissected and frozen on dry ice for analysis by Western blot. Tissues were homogenized in ice-cold RIPA buffer (FUJIFILM Wako, Tokyo, Japan) and centrifuged. Samples (20 μg of total protein) were loaded onto a 10% sodium dodecyl sulfate-polyacrylamide gel and transferred onto polyvinylidene fluoride membranes (GE Healthcare, Buckinghamshire, UK). After blocking with 3% skim milk, the membranes were allowed to react with following antibodies: anti-phospho-ERK1/2 (#9101, RRID: AB_331646), anti-ERK1/2 (#9102, RRID: AB_330744) (Cell Signaling Technology, Beverly, MA, USA), and anti-β-actin (A2228, RRID: AB_476697, Sigma, St Louis, MO, USA). After reacting with secondary antibodies (horseradish peroxidase-coupled anti-rabbit (#7074) and anti-mouse (#7076); RRID: AB_2099233 and AB_330924, Cell Signaling Technology), proteins were detected using a Luminata Forte (Merck Millipore, Nottingham, UK). The amount of detected proteins were measured based on densitometry by a CS analyzer (ATTO, Tokyo, Japan) and detected proteins were standardized to the corresponding proteins.
